# Non-Surgical Treatments for Rotator Cuff Tendinopathy: A Narrative Review

**DOI:** 10.3390/jfmk11030294

**Published:** 2026-07-26

**Authors:** Silviya P. Nikolova, Altsek Naydenov

**Affiliations:** 1 Department of Social Medicine and Healthcare Organization, Medical University—Varna, 55 Marin Drinov Street, 9000 Varna, Bulgaria; 2 Department of Kinesitherapy, Faculty of Public Health, Medical University—Varna, 55 Marin Drinov Street, 9000 Varna, Bulgaria; al.naydenov639@gmail.com

**Keywords:** rotator cuff tendinopathy, shoulder pain, exercise therapy, platelet-rich plasma, extracorporeal shockwave therapy, physiotherapy, conservative treatment

## Abstract

**Background**: Rotator cuff tendinopathy is a common musculoskeletal disorder associated with shoulder pain, functional limitations, and reduced quality of life. A wide range of non-surgical interventions have been investigated, and uncertainty remains regarding their comparative effectiveness. **Objective**: This study aimed to summarize recent evidence on non-surgical treatments for rotator cuff tendinopathy and evaluate their reported clinical outcomes. **Methods**: A structured narrative review was conducted using Google Scholar to identify studies published between 2020 and 2025 involving adult patients with rotator cuff tendinopathy. Studies investigating exercise therapy, platelet-rich plasma (PRP), extracorporeal shockwave therapy (ESWT), laser therapy, kinesiotaping, and percutaneous electrolysis were considered. Following screening and eligibility assessment, 21 studies met the inclusion criteria. **Results**: Exercise therapy was the most extensively investigated intervention and consistently improved pain, function, and shoulder mobility. PRP injections were associated with favorable effects on pain and functional outcomes, although treatment protocols varied substantially. Several studies reported improvements following ESWT in pain reduction and functional recovery. Evidence from a limited number of studies suggests that high-power laser therapy may improve clinical outcomes, whereas evidence supporting low-level laser therapy remained inconclusive. Kinesiotaping and percutaneous electrolysis demonstrated potential benefits, although the available evidence remains limited. **Conclusions**: Exercise therapy remains the cornerstone of conservative management for rotator cuff tendinopathy. Adjunctive interventions, including PRP, extracorporeal shockwave therapy, and high-power laser therapy, may provide additional benefits in appropriately selected patients. However, methodological heterogeneity and differences in treatment mechanisms limit direct comparisons between modalities.

## 1. Introduction

Rotator cuff tendinopathy is a common cause of shoulder pain and functional limitation in adults and is frequently encountered in musculoskeletal and rehabilitation practice [1]. Patients often experience pain during overhead activities, reaching movements, lifting tasks, and other activities of daily living, which may lead to reduced physical function and decreased quality of life [2].

The condition is considered multifactorial in origin. Current evidence suggests that tendon degeneration, repetitive mechanical loading, age-related tissue changes, altered vascularity, and biomechanical dysfunction may all contribute to symptom development [3,4,5]. In addition, abnormalities in scapular and glenohumeral movement patterns have been associated with increased tendon stress and persistent symptoms (p. 544, [5,6]).

Over the past decade, the management of rotator cuff tendinopathy has shifted increasingly toward conservative treatment strategies. Exercise-based rehabilitation remains the most widely recommended intervention; however, a variety of adjunctive treatments have also been investigated, including extracorporeal shockwave therapy, platelet-rich plasma injections, laser therapy, kinesiotaping, dry needling, and percutaneous electrolysis [1,7]. While many of these approaches have demonstrated promising results, the available evidence remains inconsistent, particularly regarding long-term outcomes and comparative effectiveness [7,8].

As the number of studies examining non-surgical management options continues to increase, an updated overview of the available evidence is warranted. Therefore, the aim of this narrative review is to summarize recent literature on non-surgical treatment methods for rotator cuff tendinopathy and to discuss their reported effects on pain, shoulder function, and clinical outcomes.

## 2. Materials and Methods

### 2.1. Study Design

This study was conducted as a structured narrative review to summarize recent evidence on the non-surgical management of rotator cuff tendinopathy. To improve the transparency of the review process, a predefined search strategy, explicit eligibility criteria, independent study selection by two reviewers, and standardized data extraction were used. The review was intended to provide a narrative synthesis of the available evidence rather than a comprehensive systematic review with formal methodological quality or risk-of-bias assessment, consistent with published guidance on conducting narrative literature reviews [9,10]. The review sought to address the following question: What non-surgical treatment methods are currently used for rotator cuff tendinopathy, and what outcomes have been reported in recent literature?

### 2.2. Search Strategy

A structured literature search was conducted in Google Scholar, which was selected because of its broad coverage of peer-reviewed literature and its suitability for identifying recent studies for a narrative synthesis. The search was limited to studies published between 2020 and 2025, written in English, and involving human participants. The search parameters included keywords such as (“rotator cuff tendinopathy” OR “shoulder tendinopathy”) AND (“non-surgical treatment” OR “conservative treatment” OR “physiotherapy” OR “exercise therapy” OR “manual therapy” OR “shockwave therapy” OR “dry needling” OR “injection therapy”) NOT (“calcific” OR “rotator cuff tear”). The search duration was restricted to studies published between 2020 and 2025 in order to encompass the most current methods of treatment. The search aimed to identify studies investigating conservative treatment approaches for rotator cuff tendinopathy.

### 2.3. Eligibility Criteria

Studies were eligible for inclusion if they investigated non-surgical treatment methods for rotator cuff tendinopathy, included adult participants (≥18 years), were published between January 2020 and December 2025, and were available in English. Because evidence for several conservative treatment modalities remains limited, a broad range of study designs was considered to provide a comprehensive overview of the available literature. Accordingly, randomized controlled trials, systematic reviews, meta-analyses, observational studies, clinical studies, and case reports were eligible for inclusion.

Some eligible studies included participants with supraspinatus tendinopathy or broader shoulder tendinopathy diagnoses. These studies were included when the investigated condition was clinically consistent with rotator cuff tendinopathy and the intervention was relevant to the scope of this review. Throughout the manuscript, the terminology used in the original studies has been retained where appropriate.

Studies were excluded if they focused on rotator cuff tears, investigated calcific tendinopathy, examined post-surgical rehabilitation, involved animal subjects, or if the full text could not be accessed.

### 2.4. Study Selection Process

**Figure 1:**
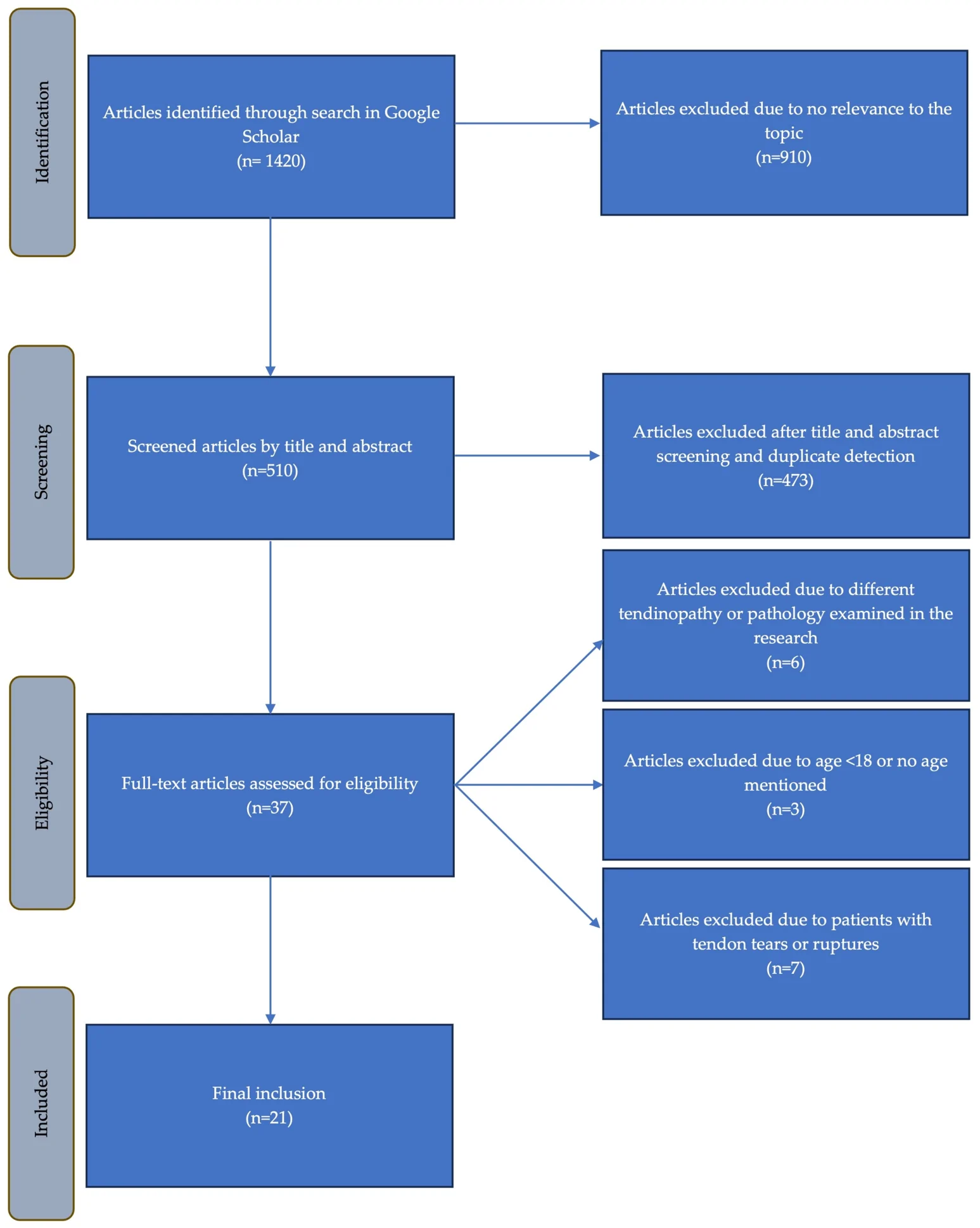
Article selection process.

Following the literature search, records were imported into Rayyan platform (Rayyan Systems Inc., Cambridge, MA, USA) for screening and study management [11]. Study selection was performed independently by two reviewers in two stages. First, titles and abstracts were screened to identify potentially relevant studies. Subsequently, full-text articles were assessed according to the predefined eligibility criteria. Any disagreements regarding study inclusion were resolved through discussion and consensus between the two reviewers. The literature search yielded 1420 records. Following an initial assessment of relevance, 910 articles were excluded. The remaining 510 studies underwent title and abstract screening, resulting in the exclusion of 473 records because they did not meet the review objectives or were duplicates. A total of 37 full-text articles were assessed for eligibility. Of these, 16 were excluded because they investigated diagnoses other than rotator cuff tendinopathy, included participants younger than 18 years of age or did not report participant age, or focused on rotator cuff tears or ruptures. Ultimately, 21 studies met the eligibility criteria and were included in the review. The study selection process is presented in Figure 1.

### 2.5. Data Extraction

Data were extracted independently by two reviewers using a standardized data extraction form. The extracted information included author and year of publication, country, study design, population and diagnosis, sample size, intervention, comparator, outcome measures, and main findings. The characteristics of the included studies are presented in Table 1, whereas the principal findings and their clinical implications are summarized in Table 2.

## 3. Results

### 3.1. Characteristics of Included Studies

A total of 21 studies met the eligibility criteria. The included evidence comprised randomized controlled trials, systematic reviews, meta-analyses, observational studies, clinical studies, qualitative studies, and case reports. Given this diversity, findings are presented narratively according to intervention type rather than by level of evidence. When interpreting the findings, greater emphasis was placed on evidence derived from randomized controlled trials, systematic reviews, and meta-analyses, while findings from observational studies and case reports were interpreted more cautiously. The studies were conducted across multiple countries, including Pakistan, China, India, Spain, the United Kingdom, Brazil, Australia, Sweden, Iran, Italy, the United States, Switzerland, Taiwan, and the United Arab Emirates. The majority of studies investigated exercise-based rehabilitation, platelet-rich plasma (PRP) injections, extracorporeal shockwave therapy (ESWT), laser therapy, kinesiotaping, and percutaneous electrolysis. Several studies evaluated these interventions as stand-alone treatments, whereas others examined them in combination with physiotherapy or exercise programmes. Sample sizes varied considerably across studies, ranging from single-patient case reports to systematic reviews and meta-analyses including multiple randomized controlled trials. Most studies involved patients diagnosed with rotator cuff tendinopathy or supraspinatus tendinopathy, while a small number included broader populations with shoulder tendinopathy or shoulder impingement syndrome. The characteristics of the included studies are presented in Table 1.

### 3.2. Exercise Therapy

Exercise therapy was among the most frequently investigated interventions identified in the included studies. Improvements in pain, shoulder function, and range of motion were reported across different exercise-based programmes. In a randomized controlled trial, therapeutic exercise resulted in significant reductions in pain and improvements in shoulder mobility, with outcomes comparable to those achieved through myofascial trigger point therapy [15]. These findings were supported by systematic reviews, where exercise-based rehabilitation was associated with favorable clinical outcomes in patients with rotator cuff tendinopathy [2].

The included studies examined a variety of exercise approaches. Progressive resisted exercise was reported to provide uncertain long-term benefits [20], whereas higher exercise loads were generally well tolerated by patients and considered acceptable within rehabilitation programmes [24]. These findings suggest that exercise therapy may be beneficial; however, the optimal exercise prescription, including loading intensity, frequency, and progression, remains unclear. Differences between isometric and isotonic exercise protocols have also been proposed as a potential factor influencing clinical outcomes; however, further research is required to determine their comparative effectiveness [22]. In addition, preliminary improvements in shoulder pain and range of motion were reported following a postural restoration exercise programme in a single-patient case report by Waldron et al. [5]. Despite the generally positive findings, considerable variation existed among exercise protocols with regard to exercise type, intensity, progression, and treatment duration. As reported by Dominguez-Romero et al. [3], this heterogeneity limits direct comparisons between studies and makes it difficult to identify an optimal exercise programme for rotator cuff tendinopathy.

### 3.3. Platelet-Rich Plasma (PRP)

Several studies evaluated the effectiveness of platelet-rich plasma (PRP) injections in the management of rotator cuff tendinopathy. Improvements in pain and shoulder function were consistently reported following PRP treatment. Clinical studies combining PRP with strengthening exercises or physiotherapy demonstrated reductions in pain and disability together with improvements in functional outcomes [16,18,19]. Evidence from systematic reviews and meta-analyses generally supported these findings. Roy et al. [7] reported significant short-term improvements in pain and function following PRP administration, whereas Lin et al. [8] observed sustained reductions in pain over longer follow-up periods. However, substantial heterogeneity in PRP preparation methods, injection techniques, and treatment protocols limits the generalizability of these findings.

### 3.4. Extracorporeal Shockwave Therapy (ESWT)

Three studies investigated the use of extracorporeal shockwave therapy (ESWT) in patients with rotator cuff tendinopathy. In a randomized controlled trial, Li et al. [13] reported greater long-term reductions in pain and superior functional outcomes following focused shockwave therapy compared with radial shockwave therapy. Comparable findings were reported by Romeo et al. [25], who observed improvements in pain, range of motion, activity level, and shoulder strength following ESWT treatment. Additional evidence was provided by Ranjithkumar et al. [14], who reported improvements in pain, muscle thickness, and shoulder function following a rehabilitation programme that incorporated ESWT and dynamic loading exercises.

### 3.5. Laser Therapy

Laser therapy was evaluated using both low-level and high-power treatment protocols. Maghroori et al. [21] reported significantly greater improvements in pain, range of motion, and disability when high-power laser therapy was combined with exercise compared with exercise alone. Similarly, Notarnicola et al. [23] found that multi-wavelength high-energy laser therapy produced superior outcomes compared with single-wavelength laser therapy. In contrast, the systematic review conducted by Leotty et al. [17] reported inconsistent findings regarding low-level laser therapy, highlighting variability among studies and uncertainty regarding its clinical effectiveness.

### 3.6. Kinesiotaping

Kinesiotaping was investigated in a randomized controlled trial conducted by Ayoub et al. [12]. The study evaluated the effects of therapeutic taping combined with physical therapy in patients with rotator cuff tendinopathy. Compared with physical therapy alone, the addition of therapeutic taping resulted in significantly greater improvements in shoulder pain, functional performance, range of motion, and motor function. Improvements were observed across multiple clinical outcome measures, including the Shoulder Pain and Disability Index (SPADI), suggesting enhanced rehabilitation outcomes when therapeutic taping was incorporated into the treatment programme.

### 3.7. Percutaneous Electrolysis and Dry Needling

Evidence regarding invasive physiotherapy interventions was limited. Rodríguez-Huguet et al. [4] compared percutaneous electrolysis with trigger point dry needling in patients with supraspinatus tendinopathy and reported superior short- and long-term outcomes following percutaneous electrolysis. Greater improvements were observed in pain intensity, shoulder function, and pressure pain thresholds in the electrolysis group compared with the dry needling group.

A summary of the principal findings and clinical implications of the included studies is presented in Table 2.

## 4. Discussion

The management of rotator cuff tendinopathy continues to rely predominantly on conservative treatment approaches aimed at reducing pain, restoring shoulder function, and improving quality of life. The studies included in this review demonstrated that a range of non-surgical interventions may contribute to clinical improvement, although differences in study design, intervention protocols, outcome measures, and follow-up duration complicate direct comparisons between treatment modalities.

Exercise therapy was the most frequently investigated intervention identified in this review. Improvements in pain, shoulder function, and range of motion were reported across randomized controlled trials, systematic reviews, and meta-analyses. Considerable heterogeneity was observed in exercise prescription, including differences in exercise type, loading strategies, progression criteria, treatment duration, and supervision. Similar findings have been reported in previous literature, where exercise remains the primary component of conservative management despite the lack of consensus regarding the optimal exercise protocol [26,27]. These observations highlight the complexity of exercise prescription in rotator cuff tendinopathy and support the use of individualized rehabilitation programmes tailored to patient presentation and functional goals [28].

PRP injections were associated with improvements in pain and shoulder function across both primary studies and evidence syntheses. These findings are consistent with previous literature, where PRP was associated with beneficial effects on pain and functional outcomes in patients with rotator cuff tendinopathy [29]. Nevertheless, interpretation of the available evidence remains challenging due to substantial variability in PRP preparation techniques, platelet concentration, leukocyte content, injection protocols, and comparator interventions [30,31]. Such methodological differences may contribute to the variability of reported treatment effects and continue to hinder the development of standardized recommendations for clinical practice.

Studies investigating extracorporeal shockwave therapy also reported improvements in pain, shoulder function, and activity-related outcomes. Similar findings have been described in systematic reviews examining shoulder pain and shoulder impingement disorders, where ESWT was associated with clinically meaningful reductions in pain and disability [32]. The biological effects of ESWT have been attributed to several mechanisms, including stimulation of tissue regeneration, promotion of neovascularization, modulation of inflammatory responses, and alterations in pain signaling pathways [33,34]. Nevertheless, substantial variation in treatment parameters, including shockwave type, energy flux density, treatment frequency, and follow-up duration, limits comparability across studies and may contribute to the variability of reported outcomes [34].

The findings concerning laser therapy were less consistent. While studies investigating high-power laser therapy reported favorable effects on pain, disability, and shoulder function, evidence regarding low-level laser therapy remained inconclusive. Previous observations have been reported in previous reviews, which identified uncertainty regarding the magnitude and clinical relevance of laser therapy effects in rotator cuff-related shoulder disorders [35,36]. Variability in treatment dosage, wavelength, duration of application, and patient selection may contribute to the inconsistent findings reported across the literature [36]. Furthermore, laser therapy is frequently administered as an adjunct to exercise-based rehabilitation, making it difficult to determine its independent contribution to clinical improvement [37]. Consequently, the available evidence does not currently allow definitive conclusions regarding the optimal treatment parameters or the patient populations most likely to benefit from this intervention.

Evidence regarding kinesiotaping and percutaneous electrolysis was comparatively limited. Although the available studies reported improvements in pain and functional outcomes, the evidence base supporting these interventions remains substantially smaller than that for exercise therapy, PRP, or extracorporeal shockwave therapy. Previous evidence syntheses have highlighted important limitations, including small sample sizes, methodological heterogeneity, and variability in treatment protocols [38,39]. In the case of kinesiotaping, a Cochrane review concluded that the certainty of evidence regarding improvements in pain and function remains very low, making it difficult to establish clear clinical recommendations [38]. By contrast, systematic reviews of percutaneous electrolysis have reported favorable effects on pain and function in patients with musculoskeletal disorders and tendinopathies, particularly when combined with exercise-based rehabilitation; however, the overall quality of evidence remains limited and further high-quality trials are required [39,40]. Consequently, the current evidence remains insufficient to determine the comparative effectiveness of these interventions relative to other conservative treatment approaches for rotator cuff tendinopathy.

An important consideration when interpreting the findings of this review is that the included conservative interventions differ substantially in their biological mechanisms, therapeutic targets, and intended clinical applications. Consequently, direct comparisons between treatment modalities should be interpreted with caution, as superiority of one intervention over another cannot be assumed solely on the basis of reported clinical outcomes. Instead, the selection of conservative treatment should be guided by the individual patient’s clinical presentation, including symptom severity, functional limitations, duration of symptoms, activity demands, comorbidities, and response to previous interventions. Therefore, the available evidence supports an individualized, patient-centered approach in which exercise therapy remains the foundation of management, while adjunctive interventions such as PRP, extracorporeal shockwave therapy, laser therapy, kinesiotaping, or percutaneous electrolysis may be considered according to specific clinical indications and patient characteristics.

## 5. Limitations

Several limitations should be considered when interpreting the findings of this review. First, methodological differences were observed across the included studies with respect to study design, participant characteristics, intervention protocols, outcome measures, and follow-up duration. This variability limited direct comparisons between treatment approaches and precluded quantitative synthesis of the findings. Second, the review included studies investigating a broad range of conservative interventions, many of which were represented by only a small number of publications. Consequently, the strength of evidence differed considerably across treatment modalities. In addition, the inclusion of multiple study designs, ranging from randomized controlled trials to case reports, introduced variability in the level of evidence available for different interventions.

The literature search was conducted using Google Scholar as the primary database and was restricted to studies published in English. Although Google Scholar provides extensive coverage of the scientific literature, relevant studies indexed exclusively in other databases or published in other languages may not have been identified. In addition, the narrative review methodology does not incorporate formal assessment of methodological quality or risk of bias to the same extent as systematic reviews and meta-analyses. Therefore, the findings should be interpreted as a synthesis of the available literature rather than a quantitative evaluation of treatment effectiveness. Finally, no formal assessment of methodological quality or risk of bias was performed, which may limit the strength of the conclusions drawn from the included evidence.

## 6. Future Directions

Future studies should focus on improving methodological consistency across studies investigating non-surgical management of rotator cuff tendinopathy. Standardization of intervention protocols, outcome measures, and follow-up periods would facilitate comparison of findings and strengthen the evidence base supporting clinical decision-making. This need is particularly relevant for exercise therapy, PRP injections, and extracorporeal shockwave therapy, where substantial variation in treatment parameters continues to limit interpretation of the available evidence.

Additional high-quality randomized controlled trials are required to evaluate interventions for which evidence remains limited, including kinesiotaping and percutaneous electrolysis. Future studies should also investigate patient-specific factors that may influence treatment response, such as symptom duration, tendon pathology severity, age, activity level, and psychosocial characteristics. Furthermore, greater emphasis should be placed on long-term outcomes, as many studies continue to focus primarily on short-term improvements in pain and function. A more individualized, patient-centered approach to treatment selection, based on the patient’s clinical presentation, symptom characteristics, functional goals, and response to previous treatment, may ultimately contribute to improved clinical outcomes and more efficient rehabilitation strategies for patients with rotator cuff tendinopathy.

## Figures and Tables

**Table 1:** Profile of Included Studies.

Author (Year)	Country	Study Design	Population/Diagnosis	Sample Size	Age (Mean ± SD)	Intervention	Comparator
[12]	Pakistan	RCT	Rotator cuff tendinopathy	56	Intervention group: 42.5 Control group: 43.1	Therapeutic taping + physical therapy	Physical therapy alone
[13]	China	RCT	Noncalcific rotator cuff tendinopathy	46	Group A: 50:6 ± 5:2 Group B: 53:4 ± 6:7	Focused shockwave therapy	Radial shockwave therapy
[14]	India	Pilot RCT	Noncalcific rotator cuff tendinopathy	75	18–60	ESWT, ultrasound, low-level laser therapy	Dynamic loading exercises
[15]	Spain	RCT	Rotator cuff tendinopathy	72	TE group 49.22 ± 15.29 MTP group 49.03 ± 19.12	Therapeutic exercises	Myofascial trigger point therapy
[1]	UK	Literature review	Rotator cuff tendinopathy	NR	-	Conservative interventions	NR
[16]	India	Prospective clinical study	Rotator cuff tendinitis	30	30–50 (43.3%) 50 (36.7%) Less than 30 (20%)	PRP injections + strengthening exercises	No comparator
[17]	Brazil	Systematic review	Rotator cuff tendinopathy	11 studies	-	Low-level laser therapy	Various comparators
[18]	Dubai	Case report	Mild supraspinatus tendinopathy	1	34	Ultrasound-guided PRP + physical therapy	No comparator
[7]	India	Systematic review and meta-analysis	Rotator cuff tendinopathy	30 RCTs	-	PRP injections	Placebo, corticosteroids, physical therapy, dry needling
[8]	Taiwan	Systematic review and meta-analysis	Rotator cuff tendinopathy	5 RCTs	-	PRP injections	Sham injection, physiotherapy, no injection
[19]	India	Clinical study	Rotator cuff tendinopathy	30	47.3	PRP + physical therapy	No comparator
[20]	Australia	Thesis review	Rotator cuff tendinopathy	7 studies	-	Exercise therapy	Various comparators
[2]	Sweden	Systematic review (Master’s thesis)	Rotator cuff tendinopathy	11 studies	-	Exercise therapy	Various comparators
[3]	Spain	Systematic review	Rotator cuff tendinopathy	8 studies	-	Exercise therapy	Various comparators
[21]	Iran	RCT	Shoulder impingement syndrome	41	Laser group 50.28 ± 11.72 Control group 50.16 ± 12.10	High-power laser therapy + exercise	Exercise alone
[22]	USA/Brazil	Trial protocol	Supraspinatus/infraspinatus tendinopathy	46	18–60	Isometric exercises	Isotonic exercises
[23]	Italy	Prospective observational study	Shoulder tendinopathy	30	Group A 60.7 ± 10.4 (45–75) Group B 61.7 ± 13.5 (31–87)	Multi-wavelength high-energy laser therapy	Single-wavelength laser therapy
[24]	Australia	Nested qualitative study	Rotator cuff tendinopathy	12	24–67	High-load volume exercise	Low-load volume exercise
[6]	USA	Case report	Rotator cuff tendinopathy	1	22	PRI^®^ exercises	No comparator
[25]	USA/Switzerland	Observational trial	Shoulder tendinopathy	40	G1 mean age—54.9 G2 mean age—62.75	ESWT	Physiotherapy + kinesitherapy
[4]	Spain	Single-blinded RCT	Supraspinatus tendinopathy	36	TDN 40.92 PE 39.17	Percutaneous electrolysis	Trigger point dry needling

**Table 2:** Summary of main findings and implications.

Study	Main Findings	Implications
[12]	Significant improvement in SPADI scores, ROM, and motor function was observed with therapeutic taping combined with physical therapy compared with physical therapy alone	Therapeutic taping may provide additional short-term benefits when combined with rehabilitation.
[13]	Focused shockwave therapy demonstrated greater long-term pain reduction and functional improvement than radial shockwave therapy	One randomized controlled trial suggests focused SWT may provide superior long-term outcomes compared with radial SWT.
[14]	ESWT combined with dynamic loading exercises improved pain, muscle thickness, and shoulder function	Current evidence suggests ESWT may improve rehabilitation outcomes
[15]	Both therapeutic exercise and myofascial trigger point therapy reduced pain and improved ROM	Exercise therapy may represent a cost-effective alternative
[16]	PRP injections combined with strengthening exercises improved pain and shoulder function over 12 weeks	PRP may be considered for selected patients, although evidence remains heterogeneous.
[18]	PRP combined with physical therapy resulted in marked improvement in pain and disability scores	Combined PRP and rehabilitation was associated with improved clinical outcomes in the reported case.
[7]	Meta-analysis demonstrated significant short-term benefits of PRP injections for pain and function	PRP may improve short-term pain and function
[8]	PRP demonstrated long-term pain reduction, although functional improvement remained inconsistent	PRP effectiveness may vary depending on outcome measures
[19]	PRP therapy improved pain and functional outcomes at 12 weeks	PRP was associated with improvements in pain and shoulder function, although treatment protocols varied considerably across studies
[20]	Progressive resisted exercise demonstrated uncertain long-term clinical benefit	Evidence regarding optimal exercise loading remains inconsistent
[2]	Exercise therapy appears beneficial in improving pain and function in rotator cuff tendinopathy	Exercise therapy remains the cornerstone of conservative management for rotator cuff tendinopathy
[3]	Heterogeneous findings prevented definitive conclusions regarding exercise therapy effectiveness	Further high-quality studies are needed
[21]	High-power laser therapy improved pain, ROM, and disability compared with exercise alone	High-power laser therapy may provide additional benefit when used as an adjunct to exercise, although evidence remains limited.
[17]	Systematic review findings regarding low-level laser therapy were controversial	Evidence regarding laser therapy remains inconclusive
[22]	Comparative effects between isometric and isotonic exercise protocols were investigated	Exercise type may influence rehabilitation outcomes
[23]	Multi-wavelength laser therapy demonstrated greater improvements than single-wavelength therapy	Preliminary evidence suggests multi-wavelength protocols may enhance treatment effects
[24]	High-load exercise was considered acceptable by participants with rotator cuff tendinopathy	Higher exercise loads appear feasible and acceptable in selected patients in rehabilitation
[6]	PRI^®^ exercises improved ROM and reduced shoulder pain	Evidence from a case report suggests that core-focused rehabilitation may influence shoulder outcomes
[25]	ESWT improved pain, ROM, activity level, and strength	Current evidence suggests that ESWT may improve pain and function; however, further high-quality studies are needed.
[4]	Percutaneous electrolysis demonstrated superior short- and long-term outcomes compared with dry needling	Preliminary evidence suggests percutaneous electrolysis may improve outcomes compared with dry needling.

## Data Availability

No new data were created or analyzed in this study. Data sharing is not applicable to this article.

## Abbreviations

The following abbreviations are used in this manuscript:

RCT	Randomized controlled trial
PRP	Platelet-rich plasma
ESWT	Extracorporeal shockwave therapy
SWT	Shockwave therapy
ROM	Range of motion
SPADI	Shoulder Pain and Disability Index
PRI^®^	Postural restoration institute
